# An increase in the extracellular potassium concentration can cause seizures

**DOI:** 10.1186/1471-2202-16-S1-P113

**Published:** 2015-12-18

**Authors:** Tianlin Ying, David B Grayden, Anthony N Burkitt, Tatiana Kameneva

**Affiliations:** 1 NeuroEngineering Laboratory, Department of Electrical and Electronic Engineering, University of Melbourne, Melbourne, Victoria 3010, Australia; 2 Bionics Institute, Melbourne, Victoria 3002, Australia

## Introduction

Epilepsy is a neurological disorder characterized by recurrent seizures. It is important for the development of patient treatments to understand the mechanisms underlying this complex neurological disease. Experimental data shows that an increase in the extracellular potassium concentration, [K^+^]_0_, can support the generation of seizures and growth in seizures frequency and propagation velocity [1]. It is unclear if seizures are caused by the increase in [K^+^]_0 _or seizures cause the [K^+^]_0 _increase.

## Methods

Using a single column model neural mass model of pyramidal cell population [2], we explore how the change in [K^+^]_0 _affects the model output. The model represents the activity of approximately 10^5 ^neurons and has four interacting neural populations: pyramidal neurons, excitatory, slow- and fast-inhibitory neurons. The model output is interpreted as representing the recorded EEG signal. A static nonlinear function (a sigmoid) of the model converts a postsynaptic potential into an average spiking rate for each neural population. We fit data from [3] to different forms of the sigmoid corresponding to different concentration of [K^+^]_0_. Simulations were run with different sigmoid parameters while other parameters of the model are fixed to produce normal background activity (non-seizure dynamics). To classify the model output as a particular neural activity type, a power spectral density analysis is used and the types were compared to those in [2].

## Results

Results show that a [K^+^]_0 _increase from 5 to 13 mM causes the neural dynamics to transit from Type 1 (normal background activity) to Type 6 (slow quasi-sinusoidal activity) to Type 3 (sustained discharge of spikes) to Type 1, and then to Type 5 (low-voltage rapid activity).

## Conclusions

Our results confirm that a [K^+^]_0 _increase can generate seizures. This may have implications on the development of the effective treatments for epilepsy patients. It is left for future research to investigate whether changes in [K^+^]_0 _can affect the frequency or propagation velocity of seizures. These investigations will be carried out using multiple interconnected columns.
